# Seroprevalence of anti–SARS-CoV-2 IgG antibodies in Kenyan blood donors

**DOI:** 10.1126/science.abe1916

**Published:** 2020-11-11

**Authors:** Sophie Uyoga, Ifedayo M. O. Adetifa, Henry K. Karanja, James Nyagwange, James Tuju, Perpetual Wanjiku, Rashid Aman, Mercy Mwangangi, Patrick Amoth, Kadondi Kasera, Wangari Ng’ang’a, Charles Rombo, Christine Yegon, Khamisi Kithi, Elizabeth Odhiambo, Thomas Rotich, Irene Orgut, Sammy Kihara, Mark Otiende, Christian Bottomley, Zonia N. Mupe, Eunice W. Kagucia, Katherine E. Gallagher, Anthony Etyang, Shirine Voller, John N. Gitonga, Daisy Mugo, Charles N. Agoti, Edward Otieno, Leonard Ndwiga, Teresa Lambe, Daniel Wright, Edwine Barasa, Benjamin Tsofa, Philip Bejon, Lynette I. Ochola-Oyier, Ambrose Agweyu, J. Anthony G. Scott, George M. Warimwe

**Affiliations:** 1 KEMRI-Wellcome Trust Research Programme, Kilifi, Kenya.; 2 Department of Infectious Diseases Epidemiology, London School of Hygiene and Tropical Medicine, Keppel Street, London, UK.; 3 Ministry of Health, Government of Kenya, Nairobi, Kenya.; 4 Presidential Policy and Strategy Unit, The Presidency, Government of Kenya, Nairobi, Kenya.; 5 Kenya National Blood Transfusion Services, Ministry of Health, Nairobi, Kenya.; 6 Nuffield Department of Medicine, Oxford University, Oxford, UK.

## Abstract

The spread of severe acute respiratory syndrome coronavirus 2 (SARS-CoV-2) in Africa is poorly described. The first case of SARS-CoV-2 in Kenya was reported on 12 March 2020, and an overwhelming number of cases and deaths were expected, but by 31 July 2020, there were only 20,636 cases and 341 deaths. However, the extent of SARS-CoV-2 exposure in the community remains unknown. We determined the prevalence of anti–SARS-CoV-2 immunoglobulin G among blood donors in Kenya in April–June 2020. Crude seroprevalence was 5.6% (174 of 3098). Population-weighted, test-performance-adjusted national seroprevalence was 4.3% (95% confidence interval, 2.9 to 5.8%) and was highest in urban counties Mombasa (8.0%), Nairobi (7.3%), and Kisumu (5.5%). SARS-CoV-2 exposure is more extensive than indicated by case-based surveillance, and these results will help guide the pandemic response in Kenya and across Africa.

## Pandemic progress in Kenya

By the end of July 2020, Kenya had reported only 341 deaths and ∼20,000 cases of COVID-19. This is in marked contrast to the tens of thousands of deaths reported in many higher-income countries. The true extent of COVID-19 in the community was unknown and likely to be higher than reports indicated. Uyoga *et al.* found an overall seroprevalence among blood donors of 4.3%, peaking in 35- to 44-year-old individuals (see the Perspective by Maeda and Nkengasong). The low mortality can be partly explained by the steep demographics in Kenya, where less than 4% of the population is 65 or older. These circumstances combine to result in Kenyan hospitals not currently being overwhelmed by patients with respiratory distress. However, the imposition of a strict lockdown in this country has shifted the disease burden to maternal and child deaths as a result of disruption to essential medical services.

*Science*, this issue p. 79; see also p. 27

## Teaser

By May 2020, 1 in 20 Kenyan adults had SARS-CoV-2 antibodies, when fewer than 100 COVID-19 deaths had been reported nationally.

Africa accounts for 17% of the global population (*1*) but by late July 2020 accounted for only 5% of the global COVID-19 cases and 3% of global COVID-19 deaths reported (*2*). This disparity has been attributed to limited capacity for diagnosis, timely implementation of stringent containment measures, a younger population structure, and a predominance of asymptomatic and mild infections (*3*, *4*). The first case of COVID-19 in Kenya was detected on 12 March 2020. Within 1 week, the government instituted containment measures to limit the spread of the virus (*5*). By 31 July national surveillance recorded 20,636 cases and 341 deaths (*6*). This increase in cases is notably slower than the epidemic in Wuhan, Europe, or the United States. Recently, it has been suggested that “the virus is spreading… …with an attenuated outcome in Africa” [(*7*), p. 626], but there are few data available to confirm or refute this assertion.

In countries affected early in the pandemic, serological surveillance was used to define cumulative incidence. For example, at the release of lockdown in Wuhan, 9.6% of staff resuming work were found to have antibodies to severe acute respiratory syndrome coronavirus 2 (SARS-CoV-2) (*8*). At the end of the epidemic wave in Spain, seropositivity was 5.0% in a random population sample of 60,897 (*9*). As the epidemic curve declined in Geneva, seroprevalence rose over 3 weeks from 4.8 to 10.9% (*10*). Currently, there are few estimates of SARS-CoV-2 seroprevalence in Africa in the literature (*11*).

Movement restrictions, in response to COVID-19, have limited the conduct of fieldwork for population-based serosurveys. Several countries have monitored seroprevalence in blood transfusion donors (*12*, *13*) or expectant mothers attending antenatal clinics (*14*). Here, we report the results of a pragmatic national serosurvey using residual blood samples from transfusion donors across Kenya and a highly sensitive and specific assay for anti–SARS-CoV-2 spike immunoglobulin G (IgG).

We validated a widely used enzyme-linked immunosorbent assay (ELISA) for SARS-CoV-2 IgG (*15*) with 910 serum samples from the prepandemic period and 174 sera from polymerase chain reaction (PCR)–defined SARS-CoV-2 cases, and a well-characterized five-sera panel from the National Institute of Biological Standards and Control (NIBSC) in the UK. For either receptor-binding domain (RBD) or whole spike, specificity was higher when using a ratio of the sample optical density (OD)/negative control OD than when using the raw sample OD plus 3 standard deviations to define seropositivity (table S1). By using OD ratios, both RBD and spike ELISAs correctly classified 901 of 910 prepandemic samples as seronegative (table S1). However, the spike ELISA detected more seropositives (166 of 179 compared with 145 of 179 for RBD ELISA) among sera from SARS-CoV-2 PCR-positive individuals (fig. S2, A and B). On the basis of these data, we defined anti–SARS-CoV-2 IgG seropositivity as an OD ratio >2 and selected the spike ELISA for this study. The sensitivity and specificity, at this threshold, were 92.7% [95% confidence interval (CI), 87.9 to 96.1%] and 99.0% [95% CI, 98.1 to 99.5%], respectively (figs. S3, A and B, S5, and S6; and table S1). As previously noted (*15*), the RBD and whole-spike ELISA responses were highly correlated (fig. S3C), with very little interassay variation (fig. S4).

**Fig. 1:**
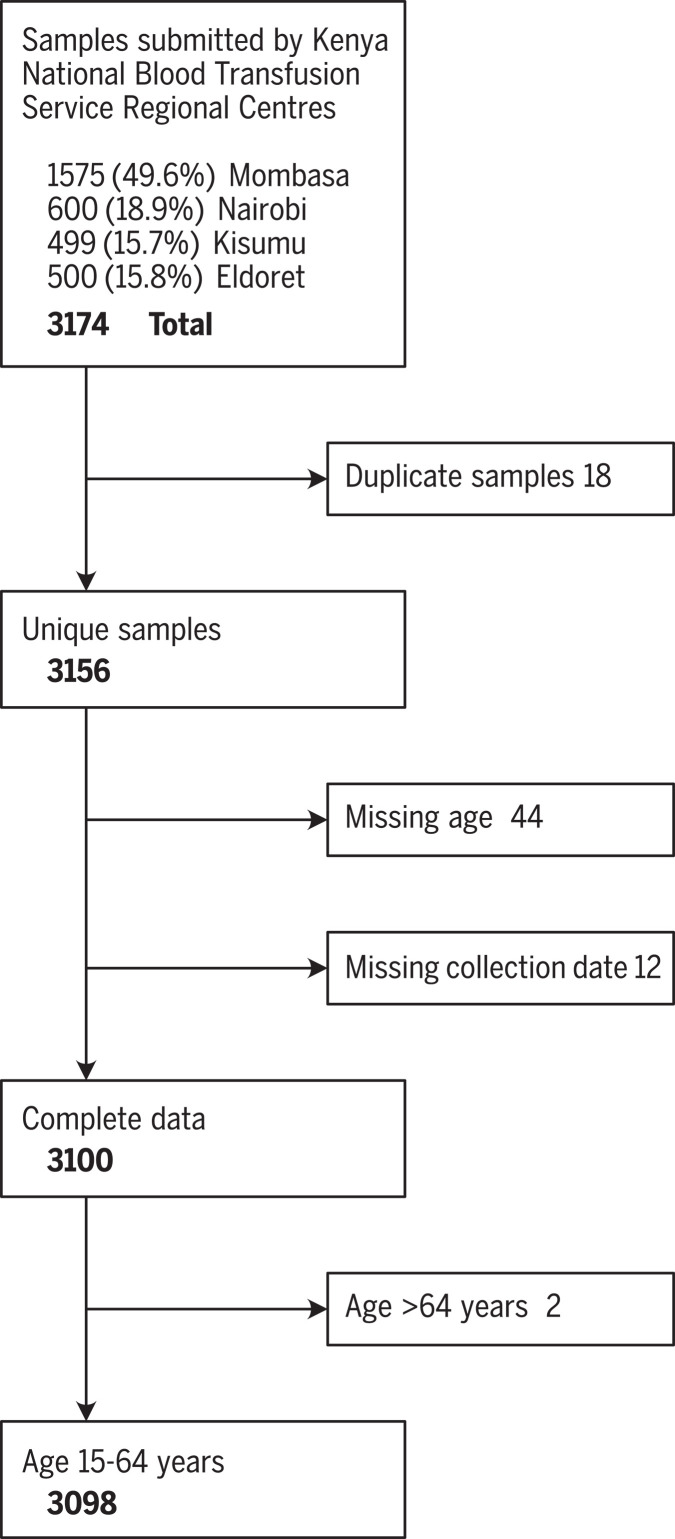
Participant flow diagram for SARS-CoV-2 seroprevalence study of blood donors in Kenya. Exclusion criteria for the selection of samples with complete data.

A total of 3174 blood transfusion samples were collected from four Kenya National Blood Transfusion Service (KNBTS) regional blood transfusion centers that are supported by several satellites and hospitals between 30 April and 16 June 2020, from individuals aged 15 to 66 years. Approximately half of the samples were drawn in Mombasa; the remainder were evenly distributed between Nairobi, Kisumu, and Eldoret (Fig. 1 and table S2). We excluded 18 duplicate samples, 56 records missing data on age or collection date, and two records from individuals aged ≥65 years. Policy in Kenya is to avoid blood donation from individuals >65 years, and we excluded these other data points as potentially unreliable. These exclusions left 3098 samples for further analysis (Fig. 1).

Of the 3098 samples, 174 were positive for anti–SARS-CoV-2 spike IgG, giving a crude seroprevalence of 5.6% (95% CI, 4.8 to 6.5%). Crude seroprevalence varied by age (*P* = 0.046), ranging between 3.4 to 7.0% among adults 15 to 54 years; all 71 donors aged 55 to 64 years were seronegative (Table 1). Crude seroprevalence did not vary by sex (*P* = 0.50) but did vary geographically, from 1.9% in the Rift Valley region to 10.0% in the Western region (*P* = 0.002) (Table 1).

**Table 1:** Crude, population-weighted, and test performance–adjusted SARS-CoV-2 anti-spike protein IgG seroprevalence by participant characteristics and regions. Prevalence estimates were calculated by using multilevel regression and poststratification (MLRP) to account for differences in the sample population and the national population and subsequently adjusted for assay sensitivity and specificity.

	**All samples**	**Seropositive** **samples**	**Crude** **seroprevalence**		**Kenya** **population** **(2019 Census)**	**Bayesian** **population-weighted** seroprevalence*		**Bayesian** **population-weighted,** **test-adjusted** seroprevalence*	
			**%**	**(95% CI)**		**%**	**(95% CI)**	**%**	**(95% CI)**
Age									
15 to 24 years	808	49	6.1	4.5 to 7.9	9,733,174	5.1	3.7 to 6.9	4.4	2.7 to 6.4
25 to 34 years	1242	66	5.3	4.1 to 6.7	7,424,967	4.9	3.6 to 6.4	4.2	2.8 to 6.0
35 to 44 years	714	50	7.0	5.2 to 9.1	4,909,191	5.9	4.3 to 8.1	5.2	3.3 to 7.7
45 to 54 years	263	9	3.4	1.6 to 6.4	3,094,771	3.8	1.9 to 6.0	3.0	1.1 to 5.4
55 to 64 years	71	0	0		1,988,062	3.4	0.7 to 6.2	2.9	0.7 to 5.7
Sex									
Male	2540	146	5.7	4.9 to 6.7	13,388,243	4.4	2.9 to 6.2	3.6	1.9 to 5.8
Female	558	28	5.0	3.4 to 7.2	13,761,922	5.5	4.4 to 6.8	4.8	3.5 to 6.4
Regions									
Central	105	7	6.7	2.7 to 13.2	3,452,213	5.6	2.9 to 10.0	4.9	1.9 to 9.7
Mombasa	550	51	9.3	7.0 to 12.0	792,072	8.3	6.1 to 10.9	7.8	5.4 to 10.8
Other Coast	973	39	4.0	2.9 to 5.4	1,671,097	3.7	2.6 v 5.1	2.9	1.6 to 4.6
Eastern/N. Eastern	242	11	4.5	2.3 to 8.0	5,176,080	4.3	2.5 to 7.0	3.5	1.4 to 6.6
Nairobi	235	21	8.9	5.6 to 13.3	3,002,314	7.6	4.9 to 11.2	7.1	4.2 to 11.2
Nyanza	442	30	6.8	4.6 to 9.5	3,363,813	6.0	4.2 to 8.4	5.2	3.1 to 7.9
Rift Valley	481	8	1.7	0.7 to 3.3	7,035,581	2.1	1.1 to 3.6	1.5	0.4 to 3.1
Western	70	7	10.0	4.1 to 19.5	2,656,995	7.0	3.5 to 13.1	6.3	2.5 to 13.1
Total	3,098	174	5.6	4.8 to 6.5	27,150,165	4.9	3.9 to 6.2	4.3	2.9 to 5.8

*Reweighted prevalence estimates based on demographic data from the 2019 Kenya Population and Housing Census

Compared with the 2019 Kenya Population and Housing Census, our participants were more commonly male (82.0% in our study versus 49.3% in the census), had more persons aged 25 to 34 years (40.1 versus 27.3%), and more residents of coastal counties (49.2 versus 9.1%) (Table 2). We therefore adjusted the prevalence estimate for the demographics of the sample using poststratification, and for the sensitivity and specificity of the test.

**Table 2:** General characteristics of the study population compared with the national population of Kenya. *N* is the number of individuals in each stratum.

		**Blood transfusion samples**		**Kenya National Census 2019**	
		***N***	**%**	***N***	**%**
Age	15 to 24 years	808	26.1	9,733,174	35.8
	25 to 34 years	1,242	40.1	7,424,967	27.3
	35 to 44 years	714	23.0	4,909,191	18.1
	45 to 54 years	263	8.5	3,094,771	11.4
	55 to 64 years	71	2.3	1,988,062	7.3
Sex	Male	2540	82.0	13,388,243	49.3
	Female	558	18.0	13,761,922	50.7
Regions	Central	105	3.4	3,452,213	12.7
	Mombasa	550	17.8	792,072	2.9
	Other Coast	973	31.4	1,671,097	6.2
	Eastern/Northeastern	242	7.8	5,176,080	19.1
	Nairobi	235	7.6	3,002,314	11.1
	Nyanza	442	14.3	3,363,813	12.4
	Rift Valley	481	15.5	7,035,581	25.9
	Western	70	2.3	2,656,995	9.8
Total	Kenya 15 to 64 years	3098		27,150,165	

The Bayesian population-weighted and test-adjusted seroprevalence for Kenya was 4.3% (95% CI, 2.9 to 5.8%) (Table 1), and the posterior sensitivity and specificity estimates were 92.4% (95% CI, 88.0 to 95.6%) and 98.9 (95% CI, 98.2 to 99.5%), respectively. Seroprevalence was higher (4.2 to 5.2%) in the younger age groups (15 to 44 years) and declined in the older age groups (45 to 64 years) but was similar for both sexes. Seroprevalence was highest for those living in Mombasa, Nairobi, and the Western region, although the number of observations for the Western region was small. The directly standardized seroprevalence estimates are presented in table S3. Seroprevalence was also calculated for counties that had at least 120 donors sampled. The three largest urban counties of Mombasa, Nairobi, and Kisumu had SARS-CoV-2 seroprevalence of 8.0% (95% CI, 5.5 to 11.1%), 7.3% (95% CI, 4.2 to 11.4%), and 5.5% (95% CI, 2.8 to 9.6%), respectively (table S4).

**Fig. 2:**
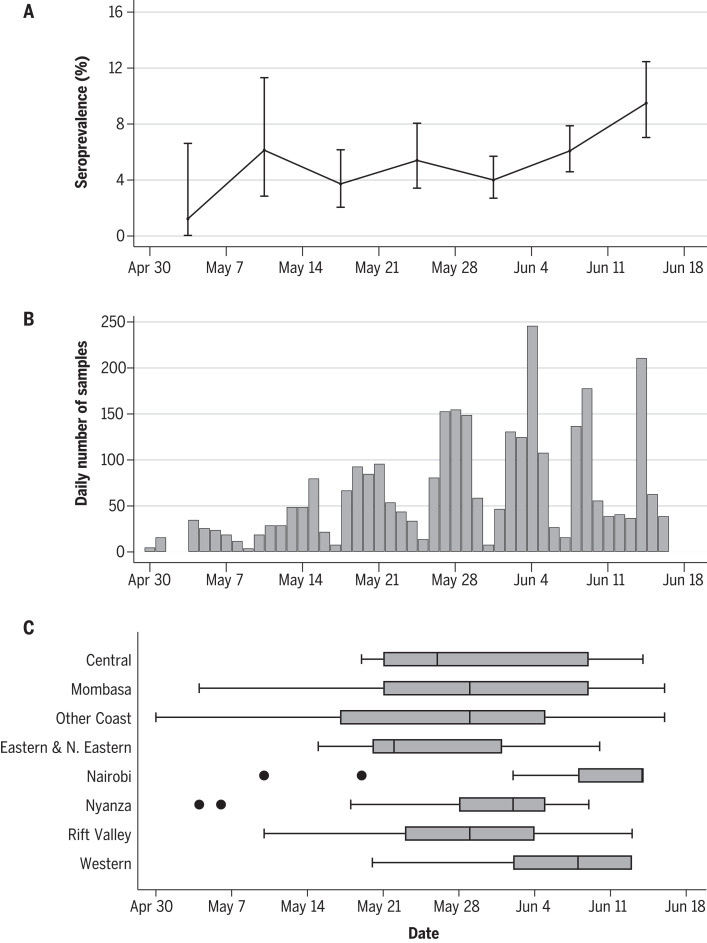
Timeline of sampling for SARS-CoV-2 seroprevalence in blood donors in Kenya. (**A** to **C**) Against the timeline of the sampling period, (A) is the weekly crude seroprevalence and 95% confidence interval, (B) is the daily frequency of samples collected, and (C) is the temporal distribution of samples by region. Shown are the proportion, counts, and regional distribution of donors during the study period.

The frequency of blood donor sampling and crude seroprevalence estimates increased with time over the 7-week study period (Fig. 2). The median sample date was 30 May 2020, and the midpoint of the study was 24 May 2020. We did not adjust for sample date because the period of sampling varied for residents of different counties (Fig. 2C); instead, we show the variation in crude prevalence over time (Fig. 2A).

Voluntary nonremunerated donors (VNRDs), who donate blood at community-based “blood drives,” made up only 7.6% (236 of 3098) of our sample of donors; the remainder were family replacement donors (FRDs) who provide a unit of blood in compensation for a transfusion received by a sick relative. The two groups did not differ significantly by age (*P* = 0.15) or sex (*P* = 0.51) (table S5). Crude seroprevalence was 8.5% (20 of 236) for VNRDs and 5.4% (154 of 2862) for FRDs. The median sample date for VNRDs (14 June 2020) was 2 weeks later than that for FRDs (29 May 2020).

Population exposure across Kenya, with a population-weighted test-adjusted seroprevalence of 4.3%, is considerably higher than was previously thought, on the basis of the cases and deaths reported to date. Seroprevalence was particularly high in the three urban counties: Mombasa (8.0%), Nairobi (7.3%), and Kisumu (5.5%). Consistent with other studies, seroprevalence did not vary significantly by sex (*9*, *10*, *16*); however, it peaked in 35- to 44-year-olds and was lowest for those ≥45 years, which is also consistent with existing reports in which seroprevalence was found to be lower in older adults (*9*, *10*).

SARS-CoV-2 seroprevalence in our study is comparable with estimates from large population-based serosurveys in China, Switzerland, Spain, and the United States after the initial epidemic peak and after many tens of thousands of deaths (*9*, *10*, *17*, *18*). Our results are also comparable with those of other surveys of blood donors in Brazil (*13*), Italy (*12*), and many parts of England (*19*). Kenya has an estimated population of 53 million in 2020, and 57% of the population is aged 15 to 64 years. If the transfusion donor seroprevalence of 4.3% was applied to all 15- to 64-year-olds, it would suggest approximately 1.3 million infections. However, by the median sample date, 30 May 2020, only 2093 cases had been detected (of which approximately 90% were asymptomatic), and there were 71 deaths among all ages (*6*). Although it is difficult to extrapolate our data directly to the whole population, they do strongly suggest that the infection is more widespread in Kenya than the current PCR test results suggest and indicate a need for more systematic testing. The current PCR testing strategy targets symptomatic individuals, health care workers, contacts of confirmed cases, international travelers, cross-border truck drivers, and residents of areas identified as “hotspots.”

What are the potential explanations for the divergence in the ratio of observed cases or deaths to serologically defined infections inferred from transfusion donors in Kenya, compared with many high-income countries? (i) The seroprevalence could be overestimated because of bias in the selection or behavior of blood transfusion donors. (ii) Cases could be underascertained by national public health surveillance, although it seems unlikely that reporting of deaths and severe cases could be reduced by several orders of magnitude, and hospitals in Kenya were not overwhelmed by admissions with respiratory illness. (iii) The steep demographic age-pyramid results in a smaller vulnerable age group. In Kenya, only 3.9% of the population is aged 65 years or greater, which is substantially less than, for example, 23.3% found in Italy; again, this would only explain a moderate reduction in severe cases or deaths (*4*). (iv) There may be alternative mechanisms of immunity to SARS-CoV-2, including cell-mediated immunity (*20*, *21*), perhaps as a result of human coronavirus (HCoV)–elicited immunity (*22*, *23*). Despite our prior work showing that HCoVs circulate in Kenya (*24*), we did not identify evidence of cross-reactive antibodies to endemic coronaviruses in our validation study.

Although blood donors are not representative of the Kenyan population as a whole, we adjusted for demographic bias in the sample structure by standardization against the age, sex, and regional distribution of the Kenyan population. A substantial proportion (43%) of the population of Kenya is outside the age range (15 to 64 years) sampled in this study, and the seroprevalence in children <15 years and adults >65 years is often lower (*9*, *10*); our estimate for blood donors may be higher than the estimate for the population as a whole. Blood donors also differ from the general population in their risk of exposure to SARS-CoV-2. For example, potential donors are excluded from giving blood if they have been ill during the past 6 months, so the sample may underestimate the population prevalence of SARS-CoV-2 antibodies; however, people who are shielding at home are unlikely to be captured in our sample, leading to an overestimate of seroprevalence. Our exploration of the two distinct populations of blood donors, FRDs and VNRDs, suggests variation in the seroprevalence by donor group, but 92% (2862 of 3098) of our sample came from the group with lower seroprevalence, and exclusion of VNRDs reduced little the crude seroprevalence in our study, from 5.6 to 5.4%. Against these considerations, other countries have relied on blood transfusion donors for an early estimate of seroprevalence, but later estimates from random population samples have not been substantially different (*25*, *26*).

A key strength of this study is the rigorous validation that included testing positive and negative control samples from the target population, as well as reference plasma from the UK NIBSC as part of a World Health Organization (WHO)–coordinated effort on SARS-CoV-2 seroepidemiology. In addition, we adopted a conservative seropositivity threshold to optimize assay specificity and sensitivity for our setting.

The pandemic response in countries with limited health care capacity has been driven by the aggressive implementation of control measures to limit transmission. Unfortunately, this strategy has been accompanied by enormous collateral costs, particularly in Africa. Modeled estimates of the disruptions of essential medical services, such as immunization and antenatal care, suggest an additional ~253,500 child deaths and 12,200 maternal deaths over 6 months in low- and middle-income countries (*27*). In the absence of social protection, the economic effects of lockdown are debilitating, so it is important to obtain an early measure of the trajectory of the epidemic.

Our study provides a national and regional estimate of population exposure to SARS-CoV-2 in an African country. The 4.3% prevalence in blood transfusion donors is in sharp contrast with the reported COVID-19 cases and deaths and supports the impression that disease may be attenuated in Africa (*7*).

## Supplementary Materials

science.sciencemag.org/content/371/6524/79/suppl/DC1 Materials and Methods Figs. S1 to S6 Tables S1 to S5 References (*29*–*32*) MDAR Reproducibility Checklist

## Appendix

View/request a protocol for this paper from *Bio-protocol*.
